# Sorcin induces gastric cancer cell migration and invasion contributing to STAT3 activation

**DOI:** 10.18632/oncotarget.22208

**Published:** 2017-10-31

**Authors:** Huan Tuo, Feng Shu, Sha She, Min Yang, Xiao Qin Zou, Juan Huang, Huai Dong Hu, Peng Hu, Hong Ren, Shi Fang Peng, Yi Xuan Yang

**Affiliations:** ^1^ Department of Infectious Diseases, The Second Affiliated Hospital of Chongqing Medical University, Chongqing 400010, China; ^2^ Institute for Viral Hepatitis of Chongqing Medical University, Chongqing 400016, China; ^3^ Key Laboratory of Molecular Biology for Infectious Diseases, Ministry of Education, Chongqing Medical University, Chongqing 400016, China; ^4^ Department of Infectious Diseases, Xiangya Hospital, Central South University, Hunan 410008, China; ^5^ Department of Health Management Center, Xiangya Hospital, Central South University, Hunan 410008, China

**Keywords:** gastric cancer (GC), sorcin, migration, invasion, iTRAQ

## Abstract

Gastric cancer (GC) is a globally occurring malignancy that is characterized by a high mortality rate due to a high tendency to metastasize and poor prognoses. Sorcin, as known as SRI, a soluble resistance-related calcium-binding protein, plays a significant role in multidrug resistance. Sorcin is related to the migration and invasion of cancer cells. However, the mechanism remains unclear. Here, we used immunohistochemistry to confirm that the expression of sorcin in cancer tissues is higher than that in the adjacent normal tissues. The wound healing and transwell results indicate that sorcin can induce migration and invasion of GC cells. To explore the role of sorcin in GC metastasis, isobaric tags for relative and absolutely quantitation (iTRAQ) were used to examine cells with and without sorcin knockdown to identify the differentially expressed proteins (DEPs). The results were evaluated via RT-PCR and western blot to confirm the ITRAQ data. Inhibition of sorcin expression can down- regulate the expression of CTSZ, MMP2, MMP9 and p-STAT3 followed by suppression of tumor growth and metastasis. Together, we concluded that sorcin has a oncogenic activity via inducing tumor growth and metastasis, leading to development of therapeutic treatments for GC.

## INTRODUCTION

Gastric cancer is the second leading cause of cancer-associated death worldwide [1, 2]. Approximately 40% of all GC cases occur in China and these are often diagnosed in the advanced stages [3, 4]. The 5 year survival rate was 28% in 2014 [5]. Many factors result in the occurrence of GC, including genetic and epigenetic alterations. In spite of this, tumor suppression genes and growth factors have been discovered which affect the progression of GC [6–8], although the molecular mechanism of tumor metastasis is still poorly documented [9].

Sorcin, a soluble resistance-related calcium-binding protein, belongs to the small penta-EF-hand protein family [10, 11]. Sorcin expressed in many human tissues and at high levels in bones, heart, brain, kidneys, breasts and skin. Sorcin has been found to over expressed in many cancers such as leukemia and gastric, ovarian and breast cancers [10, 12, 13]. Although the function of sorcin in tumors is still unclear, a number of studies have shown that sorcin may associate with multidrug resistance (MDR) [12, 14–16]. Using a gene co-expressed with P-glycoproteins in multidrug-resistant cells, sorcin was shown to be resistance-related [17]. On the other hand, sorcin is related to epithelial-to-mesenchymal (EMT) transition, which contributes to cancer metastases, as in breast cancer and colorectal cancer [14, 18].

Since sorcin plays a significant role in cancer metastasis and the mechanism for this action remains elusive, the present study utilized isobaric tags for lative and absolute quantitation (iTRAQ) to identify differentially expressed proteins (DEP) in AGS and MKN-28 gastric cancer cell lines in which sorcin had been silenced via Small interfering RNA (siRNA). After verification by PT-PCR and western blot, the DEPs were further investigated in order to identify a pathway to explain the sorcin-modulated metastatic mechanism. Our data demonstrated that sorcin can induce tumor growth and metastasis via STAT3 signaling, leading to development of therapeutic treatments for GC.

## RESULTS

### Differential expression of sorcin in tissues

**Figure 1:**
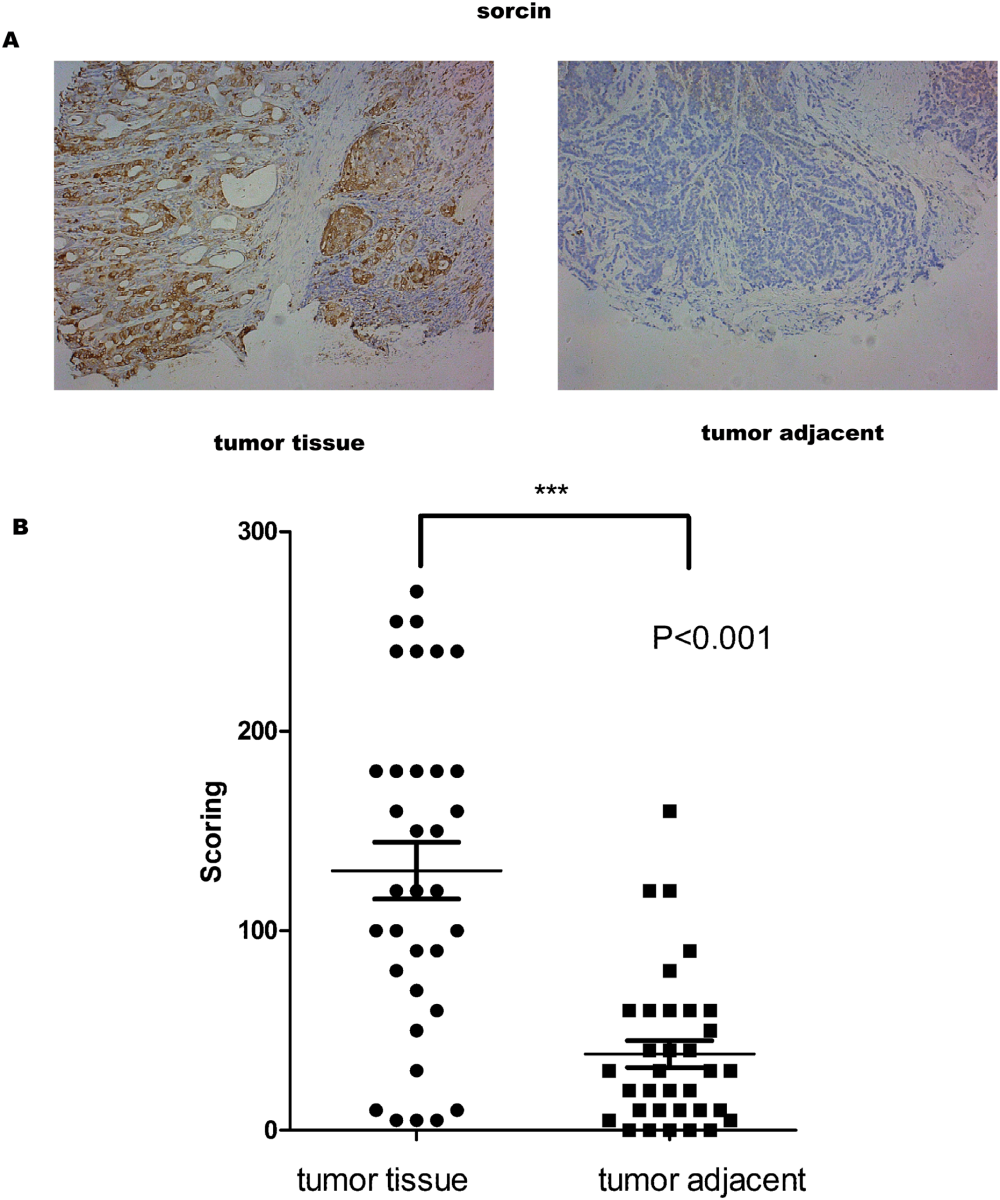
Sorcin levels were higher in GC tissue than in adjacent normal tissue **(A)** Representative images of the immmunohistochemical analysis of Sorcin in GC tissue and matched GC adjacent normal tissue. **(B)** IHC score values of Sorcin are significantly higher in GC tissue compared to matched GC adjacent normal tissue. ^*^ P<0.001.

IHC was utilized to verify whether the expression of sorcin differed between tumor tissues and adjacent normal tissues. The results showed that sorcin expression was higher in tumor tissues than that in normal adjacent tissues (Figure 1A). The IHC value of sorcin in tumor tissues was significantly higher than that in normal tissues (Figure 1B). Moreover, Pearson correlation analysis showed that IHC scoring was correlated with the Grade and Stage of tumor (Table 1).

**Table 1:** Pearson correlation coefficients between IHC scoring, tumor grade, and tumor stage

	1	2	3
1 IHC scoring	1		
2 Grade	0.817^**^	1	
3 Stage	0.869^**^	0.92^**^	1

^*^ P < 0.05 ^**^ P < 0.01 ^***^ P < 0.001.

### Effect of sorcin knockdown on migration and invasion in AGS and MKN28 cell lines

**Figure 2:**
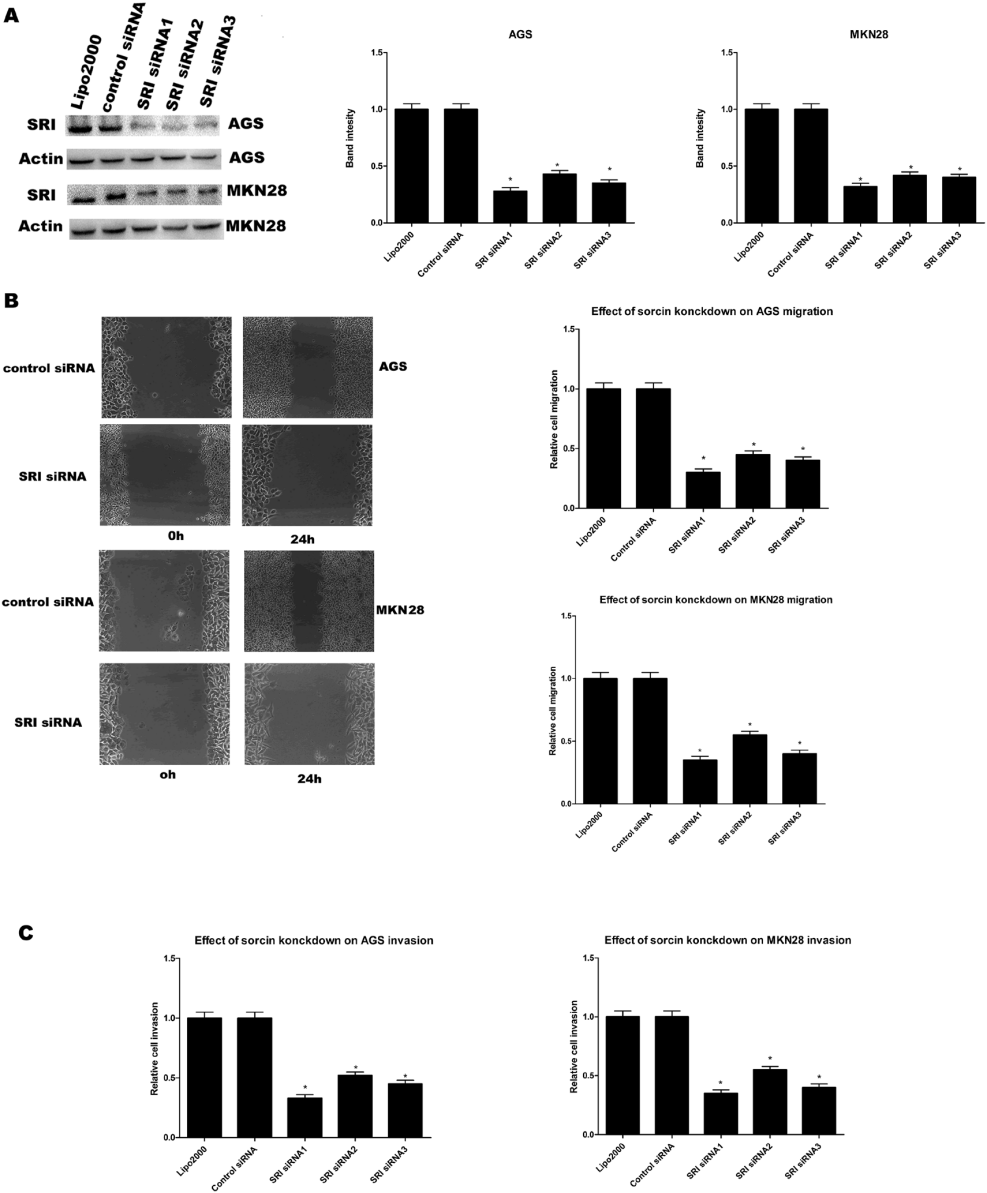
**(A)** Western blot analysis showed that three different siRNAs targeting sorcin specific siRNAs significantly reduced SRI protein levels in cell lysates, compared to control siRNA and lipo2000 and band intensity analysis**. (B)** Migration ability of cells in wound healing assays after sorcin knockdown. ^*^ P<0.05., Migration ability of AGS and MKN28 after silencing SRI. **(C)** Invasion ability of AGS and MKN28 after silencing SRI.

In order to identify the association of sorcin with GC migration and invasion, the control siRNA and SRI siRNA (SRI siRNA1 and SRI siRNA3) were transfected into the AGS and MKN28 cell lines. Cells transfected with the SRI siRNA showed significant suppression of sorcin expression (Figure 2A). Pursuant with these results, the invasion and migration capabilities of AGS and MKN28 cells were significantly decreased after transfection with SRI siRNA (Figure 2B and 2C).

### Analysis of iTRAQ data of differentially expressed proteins

The iTRAQ technology was used to explore the mechanism by which sorcin affects cell migration and invasion. The iTRAQ assays were performed on proteins isolated from AGS and MKN28 cells sorcin knockdown vs control cells without sorcin silencing. Specimens were labeled in duplicate to improve the confidence and enhance the range of protein identification. Table 2 shows the flow chart of the iTRAQ proteomics approach. The ratios of 114:113, 116:115, 118:117 and 121:119 identified the differential protein expression. An additional 1.3-fold change cutoff for all iTRAQ ratios (ratio <0.77 or >1.3) was used for classifying proteins as being up- or down-regulated.

**Table 2:** The iTRAQ-based MS workflow

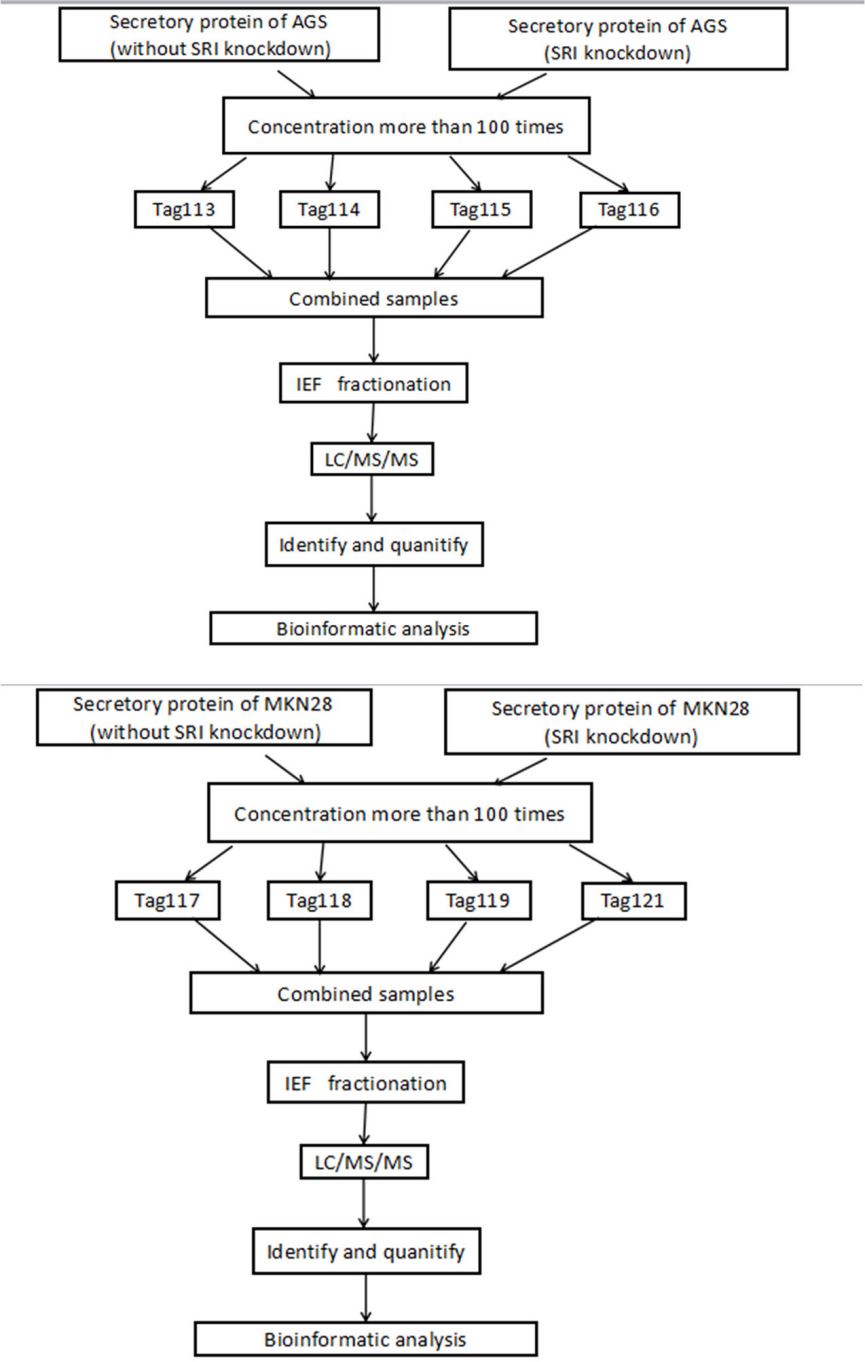

**Figure 3:**
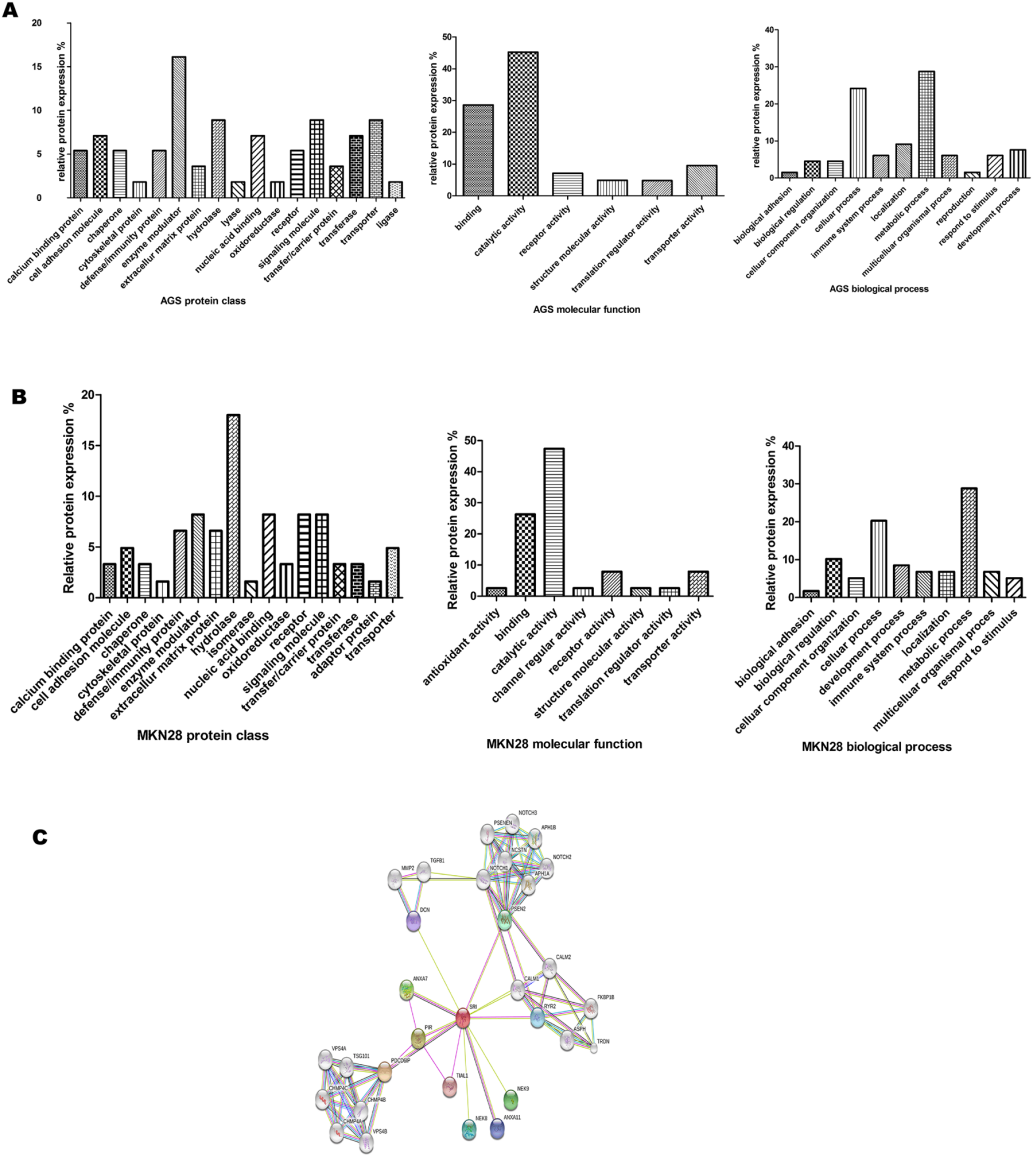
PANTHER analysis of proteins differentially expressed in GC cells **(A)** AGS Proteins were categorized by protein class, biological process, and molecular function. **(B)** MKN28 Proteins were categorized by protein class, biological process, and molecular function. **(C)** STRING analysis.

A total of 65 proteins were identified in AGS cells and 57 proteins in MKN28 cells. According to the conventions, 22 proteins were up-regulated and 43 proteins were down-regulated in the AGS line. In the MKN-28 line, 25 proteins were up-regulated and 32 were down-regulated (Table 3). To identify the cellular and molecular characteristics of these proteins, the differentially expressed proteins were classified using the PANTHER (www.pantherdb.org/) Classification System (Figure 3A and 3B). Using STRING analysis, sorcin was identified as the most important node in the DEP network because it had the greatest connectivity (Figure 3C).

**Table 3:** The proteins observed to be differentially expressed by iTRAQ analysis in supernatant of AGS and MKN28: SRI knockdown vs control

AGS						
N	Accession	Name	114:113	PVal 114:113	116:115	PVal 116:115
1	sp|Q9NPD8|UBE2T_HUMAN	Ubiquitin-conjugating enzyme E2 T	1.744066	0.026624629	1.325548983	0.044529971
2	tr|H6VRG1|H6VRG1_HUMAN	Keratin 1	1.523257017	0.001	1.7892345	0.02378564
3	sp|Q14019|COTL1_HUMAN	Coactosin-like protein	1.486366034	0.003839253	1.9871653	0.00457829
4	sp|P02787|TRFE_HUMAN	Serotransferrin	1.47558701	0.001567603	1.79888654	0.00167298
5	sp|P35527|K1C9_HUMAN	Keratin, type I cytoskeletal 9	1.456223011	0.000636358	1.89176432	0.000398717
6	tr|D3DPK5|D3DPK5_HUMAN	SH3 domain binding glutamic protein like 3	1.416821957	0.01751755	1.56279981	0.0192865
7	sp|P60174|TPIS_HUMAN	Triosephosphate isomerase	1.394827962	0.000135839	1.52817629	0.000027817
8	sp|P62195|PRS8_HUMAN	26S protease regulatory subunit 8	1.392753959	0.0281204	1.79875428	0.0367186
9	sp|Q5JS37|NHLC3_HUMAN	NHL repeat-containing protein 3	1.388023019	0.045731839	1.591827113	0.0378192
10	tr|Q53FB6|Q53FB6_HUMAN	Mitochondrial aldehyde dehydrogenase 2 variant	1.377797961	0.000768409	1.398271918	0.00036182
11	sp|P04075|ALDOA_HUMAN	Fructose-bisphosphate aldolase A	1.372123957	0.00147034	1.679281179	0.0017891
12	sp|Q5T4S7|UBR4_HUMAN	E3 ubiquitin-protein ligase UBR4	1.350811958	0.03672374	1.378291179	0.028917123
13	tr|Q6FGB3|Q6FGB3_HUMAN	PCBD protein (Fragment)	1.343956947	0.01979435	1.562919023	0.018273689
14	sp|Q9Y3C6|PPIL1_HUMAN	Peptidyl-prolyl cis-trans isomerase-like 1	1.343673944	0.01674393	1.782993739	0.017289381
15	tr|Q5U000|Q5U000_HUMAN	Cathepsin Z	0.749955177	0.035744209	0.65124789	0.02134678
16	sp|P02647|APOA1_HUMAN	Apolipoprotein A-I	0.746485889	0.01251418	0.635172839	0.013671898
17	tr|B4DVA7|B4DVA7_HUMAN	Beta-hexosaminidase	0.725379229	0.002918514	0.726019284	0.002189322
18	sp|Q0VDF9|HSP7E_HUMAN	Heat shock 70 kDa protein 14	0.725047827	0.037717941	0.6218956	0.031275433
19	sp|P18065|IBP2_HUMAN	Insulin-like growth factor-binding protein 2	0.718883276	0.007395078	0.71452389	0.001789562
20	tr|G3V1C3|G3V1C3_HUMAN	Apoptosis inhibitor 5	0.709504426	0.000174285	0.7219271	0.000342613
21	tr|Q6IBU0|Q6IBU0_HUMAN	EIF5 protein	0.707335413	0.02353386	0.682615293	0.03782919
22	sp|Q14393|GAS6_HUMAN	Growth arrest-specific protein 6	0.705515504	0.037340712	0.67281927	0.000128913
23	sp|Q96JB5|CK5P3_HUMAN	CDK5 regulatory subunit-associated protein 3	0.704363883	0.04963446	0.672918274	0.038291833
24	sp|P31949|S10AB_HUMAN	Protein S100-A11	0.67395997	0.033712041	0.631987527	0.02567891
25	sp|P23396|RS3_HUMAN	40S ribosomal protein S3	0.662013173	0.000471662	0.452719272	0.000261839
26	sp|Q9NZL4|HPBP1_HUMAN	Hsp70-binding protein 1	0.600465178	0.000367309	0.52918762	0.000271836
27	sp|P01037|CYTN_HUMAN	Cystatin-SN	0.576057613	0.001708777	0.342516829	0.001728391
28	sp|Q9H173|SIL1_HUMAN	Nucleotide exchange factor SIL1	0.574737787	0.0174674	0.328910722	0.001729372
29	sp|P21926|CD9_HUMAN	CD9 antigen	0.516185224	0.020510839	0.035617839	0.025618923
30	sp|P04080|CYTB_HUMAN	Cystatin-B	0.473518968	0.003953285	0.49178256	0.000267819

Continued MKN28						
N	Accession	Name	118:117	PVal 118:117	121:119	PVal 121:119
1	sp|P02787|TRFE_HUMAN	Serotransferrin	1.794528008	0.000295122	2.189219183	0.00028131
2	sp|Q9UNN8|EPCR_HUMAN	Endothelial protein C receptor	1.662192941	0.025397651	1.927371831	0.023618391
3	sp|Q14558|KPRA_HUMAN	Phosphoribosyl pyrophosphate synthase-associated protein 1	1.648730993	0.02934215	1.789276184	0.037826173
4	sp|Q9HAV7|GRPE1_HUMAN	GrpE protein homolog 1, mitochondrial	1.476071954	0.02040467	1.789261312	0.018927193
5	sp|P28070|PSB4_HUMAN	Proteasome subunit beta type-4	1.468814969	0.003261048	1.567289184	0.004527192
6	sp|P28070|PSB4_HUMAN	Proteasome subunit beta type-4	1.468814969	0.003261048	1.898976558	0.004261839
7	sp|O75153|CLU_HUMAN	Clustered mitochondria protein homolog	1.453933954	0.00138173	1.356183992	0.00017282
8	tr|Q6FHK7|Q6FHK7_HUMAN	PSME3 protein	1.446910977	0.007649424	1.888927368	0.001839104
9	sp|P46926|GNPI1_HUMAN	Glucosamine-6-phosphate isomerase 1	1.442389965	0.0117149	1.666618829	0.000271893
10	tr|I3L504|I3L504_HUMAN	Eukaryotic translation initiation factor 5A-1	1.413321972	0.001034902	1.789379381	0.002781939
11	tr|Q32Q12|Q32Q12_HUMAN	Nucleoside diphosphate kinase	1.385175943	0.02030622	1.89283901	0.018293134
12	tr|Q59FD4|Q59FD4_HUMAN	Hexokinase 1 isoform HKI variant	1.377347946	0.002164863	1.478290002	0.001830019
13	tr|Q6IPT9|Q6IPT9_HUMAN	Elongation factor 1-alpha	1.374544978	0.001090406	1.7829381	0.001782018
14	tr|B7Z6Q5|B7Z6Q5_HUMAN	Beta-galactosidase	0.713971794	0.01131486	0.34152719	0.027816291
15	sp|Q96AY3|FKB10_HUMAN	Peptidyl-prolyl cis-trans isomerase FKBP10	0.642570615	0.036040939	0.582981739	0.028917362
16	sp|Q06828|FMOD_HUMAN	Fibromodulin	0.640408993	0.00128549	0.527819371	0.003281903
17	sp|O00339|MATN2_HUMAN	Matrilin-2	0.633856475	0.003664825	0.608972562	0.00278183
18	sp|P36955|PEDF_HUMAN	Pigment epithelium-derived factor	0.62459141	0.032367039	0.592817293	0.034527189
19	sp|Q92930|RAB8B_HUMAN	Ras-related protein Rab-8B	0.623568416	0.017293719	0.672910381	0.019287301
20	sp|P16035|TIMP2_HUMAN	Metalloproteinase inhibitor 2	0.617994773	0.00642046	0.72341819	0.007189201
21	sp|P05121|PAI1_HUMAN	Plasminogen activator inhibitor 1	0.61286068	0.046489481	0.626152901	0.056289129
22	tr|D3DQH8|D3DQH8_HUMAN	SPARC	0.597940624	0.000163077	0.462791023	0.000179038
23	tr|Q1L857|Q1L857_HUMAN	Ceruloplasmin	0.59214592	0.003437676	0.415272801	0.002619939
24	tr|J3KPA4|J3KPA4_HUMAN	Vascular endothelial growth factor A	0.587758303	0.03545526	0.362891004	0.027810381
25	tr|Q5U000|Q5U000_HUMAN	Cathepsin Z O	0.578644204	0.026196879	0.26783619	0.002819381
26	sp|P22692|IBP4_HUMAN	Insulin-like growth factor-binding protein 4	0.57805872	0.01133533	0.703739491	0.022367192
27	tr|E7EV71|E7EV71_HUMAN	Latent-transforming growth factor beta-binding protein 1	0.531398475	0.006499027	0.462739103	0.006728913
28	sp|P13645|K1C10_HUMAN	Keratin, type I cytoskeletal 10	0.512651515	0.01043807	0.537280182	0.003781928
29	tr|C9J0K6|C9J0K6_HUMAN	Sorcin	0.43533988	0.048046826	0.50781692	0.03681903
30	sp|Q9NZL4|HPBP1_HUMAN	Hsp70-binding protein 1	0.314401984	0.045458108	0.452719301	0.035279103

### Validation of iTRAQ identified candidate proteins

**Figure 4:**
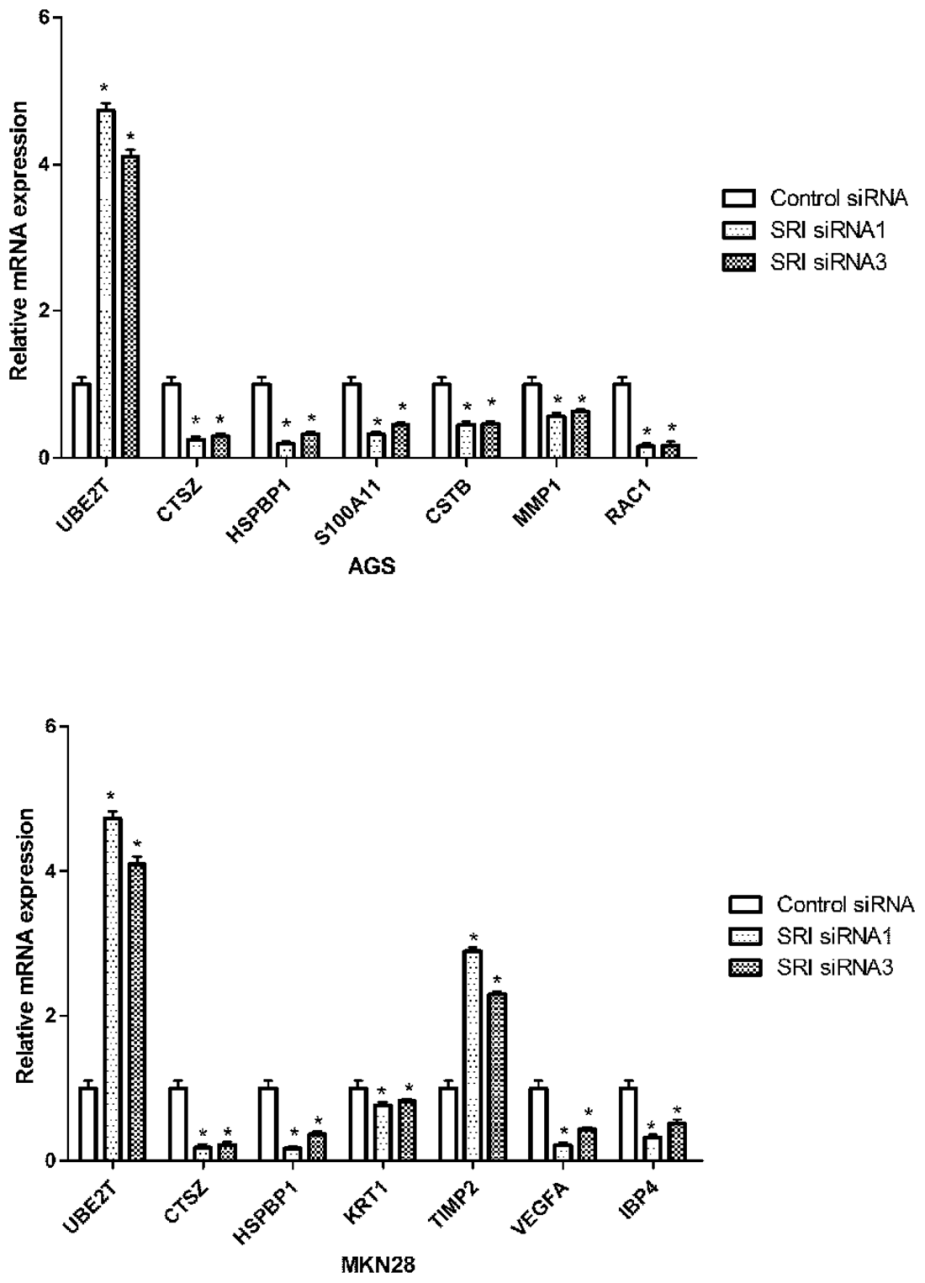
Validation of differentially expressed proteins in mRNA level RT-PCR detected the relative mRNA expression levels of some DEPs in the sorcin knockdown group compared with untreated (no knockdown) group.

**Figure 5:**
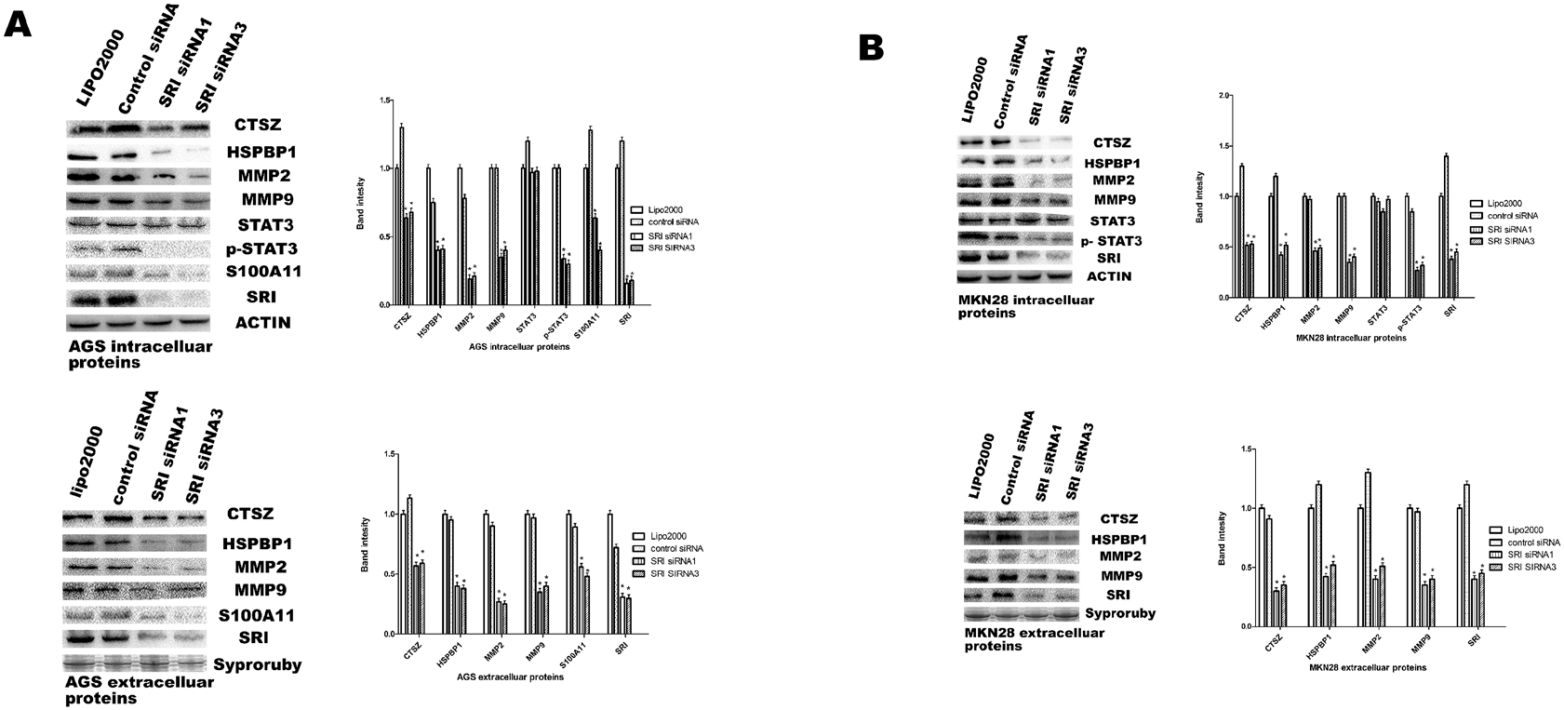
Validation of differentially expressed proteins in protein level Western blot analysis of CTSZ, HSPBP1, STAT3, p-STAT3, MMP9 and MMP2. **(A)** Protein levels from AGS cells transfected with SRI-specific or control siRNAs were analyzed by western blot analysis. Band intensity analysis shows significant reduction of p-STAT3, MMP9, and MMP2 after sorcin silencing (^*^ P<0.05). **(B)** Protein levels from MKN28 cells transfected with SRI-specific or control siRNAs were analyzed by western blot analysis. Band intensity analysis shows significant reduction of p-STAT3, MMP9, and MMP2 after sorcin silencing (^*^ P<0.05).

PT-PCR and western blot were used to confirm the changes in the selected proteins. mRNA expression of UBE2T, CTSZ, HSPBP1, S100A11, CSTB, MMP1 and RAC1 were in line with the iTRAQ resultsin AGS cells. In the MKN-28 cell line, mRNA expression of UBE2T, CTSZ, HSPBP1, KRT1, TIMP2, VEGFA and IBP4 were similar to the iTRAQ results. After silencing of sorcin, the mRNA expression of UBE2T was significantly up-regulated compared with 18s, while the mRNA expression of the other identified proteins was down-regulated (Figure 4). Western blot analysis was utilized to quantify the expression levels of the selected proteins (Figure 5A and 5B). The results of CTSZ and HSPBP1 are consistent to the iTRAQ data.

Knockdown of sorcin suppresses p-STAT3, MMP9 and MMP2 protein expression. Many studies have reported that the overexpression of p-STAT3, MMP2 and MMP9 promotes metastasis. We detected the expression of these proteins in both silenced and untreated cells. The results demonstrated that the down regulation of sorcin inhibits p-STAT3, MMP2, MMP9 expression at the protein level in AGS and MKN28 cells (p<0.05) (Figure 5A and 5B).

### Sorcin plays a role in proliferation and cell cycle

**Figure 6:**
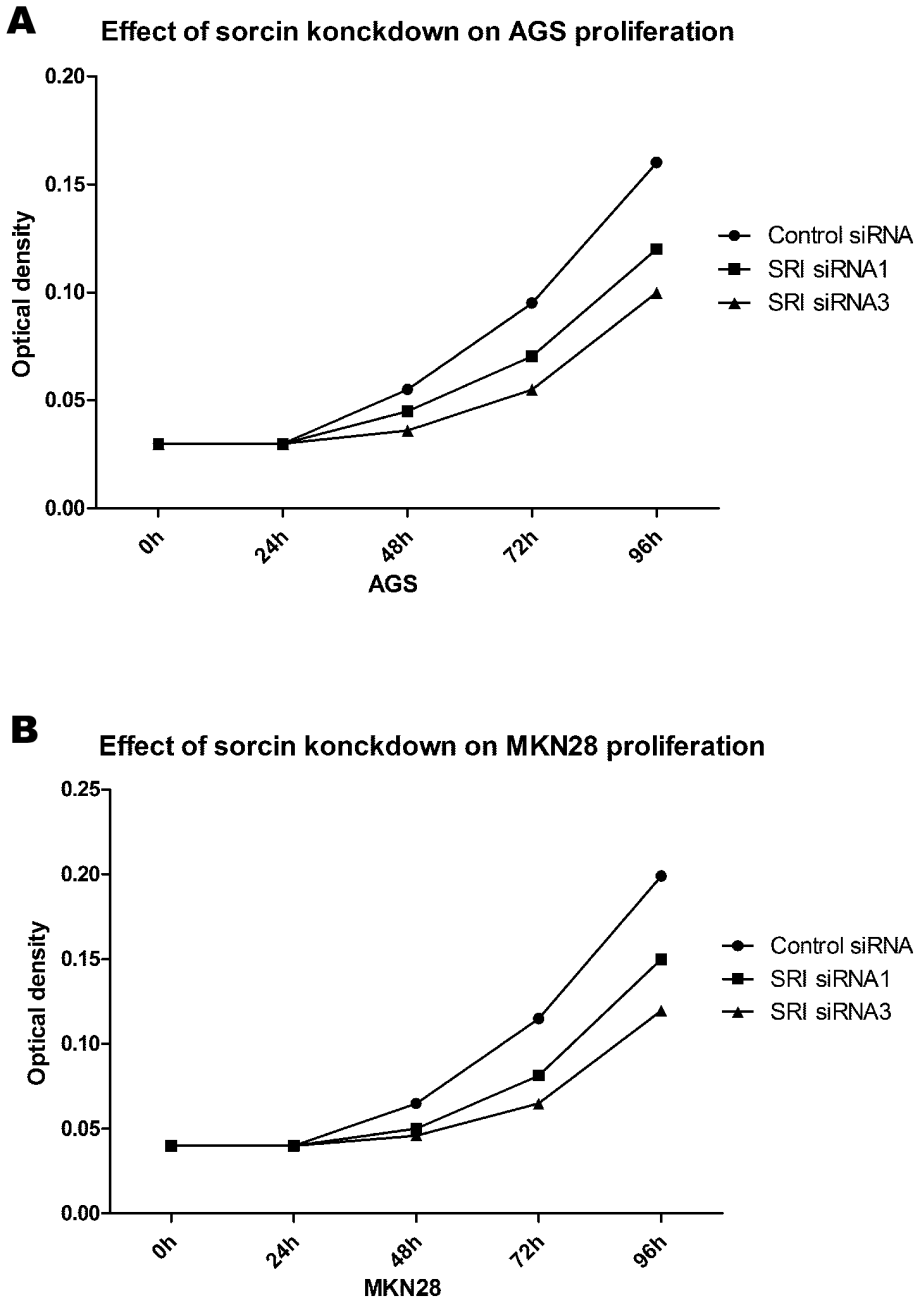
GC proliferation after knockdown of SRI **(A)** Proliferation was significant inhibit in cell transfected with SRI- specific siRNA in AGS (^*^ P<0.05). **(B)** Proliferation was significant inhibit in cell transfected with SRI- specific siRNA in MKN28 (^*^ P<0.05).

AGS and MKN28 cells were transfected with sorcin siRNA sequences. According to the western blot analysis, efficient silencing of sorcin expression was demonstrated by the sorcin-specific siRNA sequences. We also used the MTT assay to examine the proliferation of sorcin-silenced vs control AGS cells and MKN-28 cells. The proliferation of AGS and MKN28 cells was depressed compared to the control cells. We examined the change of the cell cycle and the results showed a difference in the cycles of sorcin-silenced cells compared with untreated cells (Figure 6A and 6B).

## DISCUSSION

Gastric cancer has high mortality and mobility rate and is the third leading cause of cancer deaths [19, 20]. Surgery and chemotherapy are the prime treatment for curing the malignancy [8, 20]. According to official statistics, about 50% of GC cases suffer metastases at diagnosis [21]. The 5-year survival rate is greatly decreased in patients with metastases compared with patients diagnosed early in the progression [20]. Therefore, it is urgent to explore the molecular mechanisms underlying the progression of GC, which could identify biomarkers or treatments for GC.

Sorcin is over expressed in many malignant tumors. In the present study, the iTRAQ method was utilized to compare protein expression in GC tumor tissue and compare them with expression from adjacent tissues. As previously reported, sorcin plays a vital role in invasion and migration of tumors, also has an influence on tumor proliferation.

Some of DEPs identified by the iTRAQ results were attributed to cellular processes. CTSZ, HspBp1, S100A11, CTSB, UBET2E, KRT1, MMP1, Rac1 and VEGFA were selected as candidate proteins, and PT-PCR and western blot analyses were used to validate the iTRAQ results. To identify the major changes after suppressing sorcin, we classified these proteins into different categories according to their biological process and molecular functions.

CTSZ is considered a lysosomal proteolytic enzyme and is a member of the peptide C1 family associated with many biological processes, including immune response, cell adhesion and proliferation [22]. CTSZ is over expressed in many cancers [23, 24]. CTSZ belongs to the cathepsin family of lysosomal hydrolases that contribute to the turnover of intra cellular proteins and the degradation of extracellular matrix [25]. Interestingly, several of the tumor-promoting functions of CTSZ were not dependent on its described catalytic activity [26]. Several proteases have been shown to mediate adhesion and migration of cells through interaction with integrins [27]. CTSZ contains an Arg-Gly-Asp (RGD) motif allows it to interact with integrins [26, 28]. RGD is a protein sequence vital to the binding of proteins to cell surface [29], which mediates cell adhesive properties, and this region interacts with the integrin αvb3 [30]. This interaction influences cellular migration as well as cell adhesion [30, 31]. Many studies have shown that CTSZ is related with the EMT, which could promote invasion and migration in tumor [32]. The extracellular matrix (EMT) plays an important role in modulating cell behavior, including cell survival and metastasis [33].

MMPs, a group of zinc-dependent endopeptidases, are involved in degradation of the ECM. MMP2 and MMP9 contribute to tumor metastasis [34–36]. Studies have reported signal transducer and activator of transcription 3 (STAT3) is active in many cancers [37–41]. In the present study, we found that silencing sorcin was related to metastatic activity in tumor cells. Therefore, we suspect that sorcin may be associated with the STAT3 pathway. We found that p-STAT3, MMP2, MMP9 were suppressed after silencing sorcin in GC cell lines. Taken together, these findings suggest that sorcin contribute to STAT3 activation.

In conclusion, our findings indicated that silencing sorcin affects the development of GC cells, by reducing the capacity for invasion, migration and proliferation via CTSZ and the STAT3 pathway. Therefore, suppressing the expression of the sorcin may lead to therapeutic treatments for GC.

## MATERIALS AND METHODS

### Cell lines

The two human gastric cancer lines AGS and MKN28 were obtained from ATCC, and grown in high glucose DMEM with 10% fetal bovine serum (FBS Corning), augmented with 1% penicillin and streptomycin. Cells were maintained in an incubator in an atmosphere of 5% carbon dioxide at 37°C.

### Reagents

The eight-plex iTRAQ kits were acquired from Applied Biosystems (Foster City, CA, USA). Bio-Rad (Hercules, CA, USA) provided all electrophoresis reagents. CytoSelect™ 24-well Cell Migration and Invasion assay kits (8 μm, colorimetric format) were purchased from Cell Biolabs (San Diego, CA, USA). Opti-MEM was acquired from Gibco (San Diego, CA, USA). SRI specific siRNA (HSS110179, HSS110180, HS110181) and Lipofectamine 2000 were purchased from Invitrogen (Carlsbad, CA, USA). Sorcin, CTSZ, Hspbp1 and CSTB antibody were obtained from OriGene (Rockville, MD, USA). The S100A11 antibody used in this study was purchased from Abcam (Cambridge, MA, USA).

### Sample collection and ITRAQ labeling

Cells were grown to 80% confluence, washed two times with PBS and incubated in DMEM for 24h. The supernatant containing secreted proteins was collected and filtered using a 0.22 mm filter (Millipore, Bedford, MA, USA) and concentrated by centrifugation with an Amican centrifugal filter (Billerica, MA, USA). The protein concentration was measured using a 2D Quant kit. For each sample, 100 ug of protein was precipitated overnight, re-dissolved in lysis buffer, and cysteine-blocked in accordance with the manufacturer’s instructions. Th protein samples were then labeled with the iTRAQ tags. The secretory protein samples from AGS without sorcin knockdown were labeled with tag 113 and tag 115. The secretory proteins from AGS cells with sorcin-silencing were labeled with tag 114 and tag 116. The secretory proteins from MKN-28 cells without sorcin knockdown were labeled with tags 117 and 119 and the secretory proteins from sorcin-silenced MKN-28 cells were labeled with tags 118 and 121.

### Fractionation of pepitides

The pooled iTRAQ labeled samples were dissolved in 300uL 1% Pharmalyte (Amersham Biosciences) and 8M urea and evenly coated on pre-hydrated IPG strips (pH 3-8). The peptides were isolectrically focused successively for 1h at 500v, 1h at 1000v, 1h at 3000v, and 8.5h at 8000v to give a total of 68kv in an IPGphor system (Amersham Biosciences). The peptides were extracted using an acetonitrile (ACN) and formic acid solution. Fractions were lyophilized in a vacuum concentrator and purified on an SPE C18 column. The purified fractions were re-lyophilized and stored in -20°C for use in mass spectrometric analysis.

### Mass spectrometry

The purified, labeled peptides were reconstituted in Buffer A (2% ACN; 0.1% formic acid) and injected into the nano LC ESI MS/MS system. Mass spectrometry was performed using a Qstar Elite mass spectrometer coupled to a Dionex Ultimate 3000 liquid chromatography system. A gradient series for each analysis was loaded on C-18 pepMap column at a flow rate of 0.3ul/min. The mass spectrometer was set to perform data acquisition in the positive ion mode, with a selected mass range of 300-1800 m/z. The two most abundantly charged ions above 20 counts were selected for MS/MS. Dynamic exclusion criteria was set to 30s with a ± 50 Da mass tolerance.

Protein Pilot software (version 2.0 applied biosystems, MDS Sciex) was used for the identification and qualification of proteins, with the search being carried out against the International Protein Index (IPI) human database (version 3.77).

Immunohistochemistry and tissue Microarray IHC assessment of sorcin was performed using a commercial tissue microarray (Biomax, Rockville, MD, USA) containing 40 GC samples and 40 matched GC adjacent normal tissue. Immunohistochemistry was performed on the tissue microarrays as previously described [42]. The tissue samples were dewaxed with xylene and rehydrated using an alcohol gradient. The samples were washed three times in double distilled H_2_O (5 min per wash) and subjected to heat-induced antigen retrieval in a 0.1 M citrate solution for 5 minutes. Endogenous peroxidase activity in the samples was quenched by incubation in 3% H_2_O_2_. The sections were blocked with BSA for 0.5h and incubated with anti-sorcin antibody overnight at 4°C. Detection was performed on an Envision/horseradish peroxidase system (DakoCytomation, Glostrup, Denmark) and all slides were counterstained with Gill hematoxylin for examination. Protein expression was evaluated by recording staining intensity (scale 0-3) and the percentage of positive cells (0-100%). Multiplying these scores yielded a value of 0-300. All data analyses were performed with SPSS v.16.0 software (SPSS, Chicago, IL, USA) using the student the t-test with 95% confidence levels.

### Sorcin siRNA transfection, wound healing, cell migration and invasion assays

AGS and MKN28 cells were transfected with 50nm of sorcin specific Stealth Selected RNAi siRNA (HSS) or a negative control siRNA using Lipofactamin 2000, according to the manufacturers protocol (Life Technologies, Carlsbad, CA, USA). The cells were cultured in serum-free, high-glucose DMEM with free serum for 2 days in 6-well plates. When the cells became confluent, a scratch was made in the monolayer with a sterile P200 pipette tip. The cells were washed twice with PBS to remove debris and the wound channel was photographed under a phase-contrast microscope at 0h and 24h for comparison. Adobe Photoshop 7.0 was used to measure the relative width of the wound, and cell migration was determined by the ability of the cells to close the scratch area. Migration and invasion assays was performed using Cell Invasion Assay kits (Cell Biolabs, Inc., Beijing China). AGS and MKN28 cells were transfected with negative siRNA or sorcin-targeting siRNA and cultured in free serum media. Approximately 3^*^10^5^/300ul cells were seeded into the upper chambers of the Transwell chambers, and 500ul media was loaded into the lower chamber. The migration assay cells were cultured for 12h, while the invasion assay cells were cultured for 24h. The non-invasive cells were gently removed with cotton and the invading cells were stained, fixed, extracted and quantified with cyQuant GR fluorescent dye at 560 nm. The knockdown of sorcin protein expression was confirmed by western blot.

### RNA extraction and quantitative PT-RCR

Total RNA was extracted using TRIZOL Regent (Gibico-BRL, Gaithersburg, MD, USA), according to the manufacturer’s protocol. First-strand cDNA was synthesized from 2ug of total RNA using a Reverse Transcription kit (Thermo Fisher Scientific, Waltham, MA, USA). A Fast PCR kit (KAPA SYBR, MA, USA) was used to perform PT-PCR using primers for UBE2T (Hs00311058_CE), CTSZ (Hs00216567_CE), HSPBP1 (Hs00358592_CE), S100A11 (Hs00273948_CE), CSTB (Hs00220803_CE), MMP1 (Hs00111380_CE), RAC1 (Hs00368991_CE), KRT1 (Hs00444496_CE), TIMP2 (Hs00171270_CE), VEGFA (Hs00275352_CE) and IBP4 (Hs00398644_CE) (Table 4). Quantification of gene expression was calculated using the 2^-ΔΔCT^method. RT-PCR analyses were conducted in triplicate.

**Table 4:** The sequence of genes specific primers as followed

Gene	Primer sequence	Gene	Primer sequence
UBE2T	F 5-TGCGAGCTCGTAGAAATATT-3	CTSZ	F 5-CGGCCTCATGAGTACCTGTC-3
	R 5-TTGAGGGATGGTCTCCAAGC-3		R 5-GCAGTATTGGGGGATGTGCT-3
HSPBP1	F 5-CCTCTTCGCCATCTCCTGTC-3	S100A11	F 5-CTATGGCTTGCCATGACTCCT-3
	R 5-TCAACACAGAGAAGCCGTCC-3		R 5-TACAAGAAAGTTGGGCAGGTCC-3
CSTB	F 5-TCCCTGTGTTTAAGGCCGTG-3	MMP1	F 5-GCGAGCCCTCCTTTATCTCC-3
	R 5-ACACTCGCAGGTGTACGAAG-3		R 5-AGGGTCTAAAACGCCAGTCG-3
RAC1	F 5-AGGCAAACGCCCATTGGATA-3	KRT1	F 5-GGTGTCAAGTCCTCTGGTGG-3
	R 5-ACTGCGGTCACTTACCTTGG-3		R 5-ACAAGAAAGTTGGGTATCTGGT-3
TIMP2	F 5-TCAAGAGAAGTGACGGCTCC-3	VEGFA	F 5-TGCGGATCAAACCTCACCAA-3
	R 5-ACAAGAAAGTTGGGTATGGGTCC-3		R 5-CAAGAAAGTATGGGCACCGC-3
IBP4	F 5-CAAGTGCTGGTGTGTGGACC-3		
	R 5-AAGAAAGTTGGGCACTCTCGAA-3		

### Western blot analysis

Cells were lysed with lysis buffer and the concentration of the proteins was determined by a 2D Quantification kit (GE Healthcare). The proteins were separated by 12% SDS-PAGE and transfected to PVDF membranes. The membranes were blocked in BSA for 1h at room temperature, and incubated with the primary antibody (1:500 dilution) overnight at 4°C. The membranes were washed three times and incubated with HRP-conjugated secondary antibody (1:10000). The membranes were washed thrice with TBST and visualized with the ChemiDoc MP imaging system (BioRad Laboratories, Hercules, CA, USA).

### Cell proliferation assay

AGS and MKN28 cells were seeded in 96-well plates at a density of 1.5x10^3^ cells/well. Cells were cultured in DMEM with 10% FBS and transfected with SRI siRNA or control siRNA for 0, 24, 48, 72 and 96 h at 37°C. The MTT assay was performed as follows: cells were incubated with 20 μl MTT (Sigma-Aldrich, St Louis, USA) at 37°C for 4 h. The MTT substrate was then dissolved in 200 μl of DMSO (Sigma-Aldrich, St Louis, USA) for 5 min. The absorbance was measured at 570 nm.

### Statistical analysis

All experiments were performed in triplicate and the data are expresses as the mean ± standard deviation. The Student’s t-test was used to analysis the variables between two groups. Differences were considered significant when p<0.05.
